# Internet-based biosurveillance methods for vector-borne diseases: Are they novel public health tools or just novelties?

**DOI:** 10.1371/journal.pntd.0005871

**Published:** 2017-11-30

**Authors:** Simon Pollett, Benjamin M. Althouse, Brett Forshey, George W. Rutherford, Richard G. Jarman

**Affiliations:** 1 Viral Diseases Branch, Walter Reed Army Institute of Research, Silver Spring, Maryland, United States of America; 2 Global Health Sciences, University of California, San Francisco, San Francisco, California, United States of America; 3 Marie Bashir Institute, University of Sydney, NSW, Australia; 4 Institute for Disease Modeling, Bellevue, Washington, United States of America; 5 Information School, University of Washington, Seattle, Washington, United States of America; 6 Department of Biology, New Mexico State University, Las Cruces, New Mexico, United States of America; 7 Global Emerging Infections Surveillance Section, Armed Force Health Surveillance Branch, Silver Spring, Maryland, United States of America; 8 Cherokee Nation Technology Solutions, Silver Spring, Maryland, United States of America; University of Washington, UNITED STATES

## Abstract

Internet-based surveillance methods for vector-borne diseases (VBDs) using “big data” sources such as Google, Twitter, and internet newswire scraping have recently been developed, yet reviews on such “digital disease detection” methods have focused on respiratory pathogens, particularly in high-income regions. Here, we present a narrative review of the literature that has examined the performance of internet-based biosurveillance for diseases caused by vector-borne viruses, parasites, and other pathogens, including Zika, dengue, other arthropod-borne viruses, malaria, leishmaniasis, and Lyme disease across a range of settings, including low- and middle-income countries. The fundamental features, advantages, and drawbacks of each internet big data source are presented for those with varying familiarity of “digital epidemiology.” We conclude with some of the challenges and future directions in using internet-based biosurveillance for the surveillance and control of VBD.

## Introduction

Internet-based communicable disease biosurveillance was conceived in the mid-1990s, when the ProMed system was introduced to solicit, via email, reports of early outbreaks or epidemics from media, government, clinicians, and other sources and to communicate them to public health officials and other stakeholders [1]. In 2009, after exploratory efforts by those such as Eysenbach and Polgreen et al. [2], the launch of Google Flu Trends spurred a rapidly evolving field of “digital epidemiology” or “digital disease detection” [3–5]. In this review, we use these terms interchangeably with “internet-based biosurveillance” and broadly define them as methods that are (i) based on internet-derived data, (ii) do not always require an infected person to present to healthcare to obtain communicable surveillance data, and (iii) often use nonclinical or nonlaboratory proxies for disease activity.

Since the landmark Google Flu Trends paper by Ginsberg et al. [3], a variety of studies have assessed a range of internet data sources to enhance communicable disease surveillance, including (but not limited to) Google, other internet search engines, Wikipedia, Twitter, and internet newswires [6–9]. The putative advantage of real-time internet-based surveillance systems like Google Flu Trends put forth by Ginsberg et al. (and by subsequent authors of many other papers in this field) is that timely surveillance is a cornerstone of public health response to influenza and other pathogens—yet even in well-resourced regions, conventional surveillance is not real time, and there is generally a delay of at least one to two weeks before finalized data are available. Such delays may be unacceptable in public health practice, particularly in the context of an epidemic or pandemic, when early detection may improve response and control. Google Flu Trends was initially proposed to be useful for public health practice because it was initially able to predict epidemic peaks by two weeks [3].

Real-time internet-based biosurveillance methods for vector-borne diseases (VBDs) such as dengue virus (DENV), other arthropod-borne viruses (arboviruses), malaria, and Kinetoplastida have now been studied in several tropical and temperate countries [10–14]. These VBDs affect many low- and middle-income regions of the world with rapidly rising internet access but relatively limited conventional surveillance infrastructure and often delayed dissemination of VBD surveillance data to key stakeholders [15,16]. In such regions, internet-based biosurveillance methods (which are often real time and free) may thus be useful as a supplementary, timely surveillance signal to aid in public health preparedness, situational awareness, and response to VBDs, although there may be considerable limitations to their accuracy in low- and middle-income countries because of limited and/or heterogeneous internet access [15]. Despite now numerous published studies on internet-based biosurveillance for VBD, most reviews of this topic are now relatively old and do not focus on communicable diseases, let alone VBD [4,17–20].

In this narrative review, we succinctly describe the various internet data streams and their applications in biosurveillance for vector-borne viruses, parasites, and other pathogens, including Zika virus (ZIKV), DENV, other arboviruses, malaria, *Leishmania* spp., and *Borrelia* spp. (Lyme disease) in a range of settings, including low- and middle-income countries. Through these case examples, we cover the rationale for developing these methods, the types of internet data they employ, their accuracy, and their possible uses in public health practice. We comment on the strengths and limitations of existing studies in this field and conclude with some perspectives regarding the challenges and future directions in using internet-based biosurveillance for the surveillance and control of VBD.

## Methods

This narrative review leveraged a broader search review strategy developed for an ongoing systematic review and meta-analysis for internet-based biosurveillance of any pathogen. This original literature search used the MEDLINE (PubMed) database (as of February 2, 2016), in addition to EMBASE (as of February 4, 2016) and Web of Science (as of February 4, 2016). An example of the search ontology for MEDLINE is presented in the supplementary material. From these literature search results, we then restricted to those studies pertaining to the internet-based biosurveillance of any bacterial, viral, or parasitic VBD, with no restriction by country income status. To identify further digital disease literature relevant to the recent ZIKV pandemic, which emerged during and after the initial literature search, we also searched MEDLINE using the term “Zika” (current to February 2017). We also searched specific websites describing the characteristics of individual digital data sources, such as https://trends.google.com/trends/, http://www.wikipediatrends.com/, https://twitter.com/ and http://www.internetlivestats.com/twitter-statistics/. While a structured evaluation of study quality was not undertaken for this narrative review, we did adopt aspects from a review by Nuti et al. [19] and Althouse et al. [21] to identify key items of quality and usability to consider in each article reviewed, including the spatial and temporal scale of predictions, the public health relevance of the phenomenon predicted by the models, and whether or not the models were validated on a hold-out set of data (that is, on data not used to fit the model).

## Internet-based biosurveillance data sources and their applications for VBD surveillance and control

### Google-based surveillance for DENV

The morbidity and economic burden of DENV are profound and are predicted to grow because of factors such as increasing urbanization in the tropics and expansion of the geographic range of the *Aedes aegypti* and *A*. *albopictus* vectors [22]. There is clearly a need for timely surveillance for DENV response and control in order to, e.g., guide resource allocation when epidemics may exceed hospital capacity, measure the impact of public health interventions in real time, and predict when DENV activity exceeds epidemic thresholds and peaks. Unsurprisingly, then, DENV has been the focus of several Google-based VBD detection studies in a range of socioeconomic backgrounds [10,13,14,23].

The estimated daily volume of Google searches exceeds 3.5 billion [24], and such searches are logged with precise geolocation (by IP address; the IP addresses themselves are then purged and not retained) and time. From an “unbiased sample” of these searches, Google provides de-identified, normalized, region-specific trends in search activity via the Google Trends website [25]. For more common terms, this allows a weekly estimate of, e.g., the proportion of Google searches of the term “influenza” in a particular city in the United States relative to all Google searches in that week and location. Pioneering work by Ginsberg and Polgreen noted that trends in internet search terms about influenza were closely correlated with time series of influenza cases reported to public health agencies [2,3]. This led to the development of Google Flu Trends in 2009, a customized bivariate logit model containing search activity time series of multiple distinct search terms related to influenza summed into a single predictive term, thus capturing the sensitivity and specificity for a range of Google search terms [3].

This approach was soon extended to DENV with two independent, concurrent studies by Althouse et al. and Chan et al. [10,14]. Both groups fitted and validated Google search query-based DENV prediction models. Chan et al. presented a univariate prediction model (combining the search activity trends of multiple Google terms related to DENV), and Althouse et al. presented multivariate prediction models employing multiple model validation techniques. Model performance was good in both studies. Chan et al. noted their model performed well in Brazil (r = 0.99), India (r = 0.94), Singapore (r = 0.94), and Indonesia (r = 0.94) when comparing predicted DENV holdout time series data (that is, predicted trends of DENV on a prospective period of time not used for the fitting of the predictive model) to Ministry of Health (MoH) time series data [10]. Althouse et al. found holdout correlations in Singapore (r = 0.93) and Bangkok (r = 0.88) by using a support vector machine model used to predict DENV outbreaks; Singapore and Bangkok showed areas under the receiver-operator curves ranging from 0.9 to 0.99. Notably, the model by Chan et al. performed most poorly in Bolivia (r = 0.83). While Bolivia’s internet access is less than Singapore’s, which may partly explain the difference in performance between these two countries, Bolivia has higher internet access than India and Indonesia [15], and it is more likely that the moderate accuracy of Google-based DENV prediction in Bolivia was a result of the holdout data set corresponding to a small DENV epidemic compared with the external validation of the other countries [26]. After these studies, a public Google Dengue Trends (GDT) prediction tool was launched in the four countries studied by Chan et al., along with Argentina, Bolivia, Mexico, the Philippines, Thailand, and Venezuela [27], although published GDT predictions were discontinued as of mid-2015.

From the study by Chan et al., disease burden thus appeared to be a predictor of the accuracy of Google-based DENV surveillance. This was confirmed by Gluskin et al. in a study that modeled determinants of the same model’s accuracy in several diverse areas of Mexico [23], compared to MoH DENV surveillance data. An important finding of this study was that GDT’s performance often deteriorated with finer spatial resolution, that is, it often performed best when the model was applied on a national scale (all Mexico *r* = 0.91) versus the state level (with R^2^ values as low as = 0.1, although performance in some states such as Chiapas was as high as R^2^ = 0.88). It was noted that those regions with less DENV burden (e.g., the state of Chihuahua) had worse GDT performance. Temperature and precipitation (which would support a strong seasonal trend of DENV cases) were also noted to be key predictors. Intriguingly, this correlation between climate and the performance of internet-based biosurveillance has been noted in tropical regions for influenza, with a suggestion that those tropical regions with less temperate cyclical influenza activity may experience worse performance of internet-based biosurveillance, partly because of less seasonal autocorrelation [28].

This dependence of Google-based DENV surveillance accuracy on DENV burden was further supported in a study by Milinovich et al. that noted a poorer performance of Google-based DENV prediction in Australia (*r* = 0.75) despite its very high internet access [15]. While Milinovich et al. only used a single Google search term to predict DENV activity, the discrepancy between internet access and performance of Google-based DENV surveillance may reflect the low incidence of DENV in Australia, which only experiences small local outbreaks in one state (Queensland) in addition to travel-imported cases [29,30]. This study also demonstrated a breakdown of predictive accuracy with finer spatial resolution, with poorer predictions in any one state compared to those based on Australia-wide aggregated data. Such a possible dependency of Google-based DENV surveillance on larger spatial scales is potentially concerning because, ultimately, public health action is taken locally and best guided by more granular surveillance data. However, as mentioned above, the models presented by Althouse et al. showed high predictive accuracy in two individual cities—Singapore and Bangkok—at weekly and monthly resolutions, respectively [14].

Taken together, Google-based DENV surveillance appears more accurate in the setting of (i) DENV endemicity, (ii) high burdens of disease (which may also lower the threshold for populations searching about DENV-related terms when experiencing a febrile syndrome), and (iii) climates characterized by seasonal high rainfall periods resulting in seasonal or semiseasonal peaks of disease. Its performance in nonendemic settings, such as the returning traveler to a nonendemic country, seems more limited, even in the context of high internet access. While there is some evidence that it is dependent on larger temporal and spatial scales, its performance has been shown to be excellent in a single hyperendemic city with a weekly temporal resolution [14].

### Google-based surveillance for other VBD

Google search data have also been explored for enhancing the surveillance of several other arboviruses. As part of a scoping study to screen the suitability of Google-based surveillance for a range of pathogens, Milinovich et al. determined the correlation of Google search terms with monthly Australian MoH surveillance for endemic alphaviruses (Ross River virus [RRV] and Barmah Forest virus [BFV]), a nonendemic alphavirus (Chikungunya virus [CHIKV]), and a local neurotropic flavivirus (Murray Valley encephalitis [MVE]). While a robust external validation of a prediction model was not an aim of this study, it was notable that Google data performed best in the detection of arboviruses endemic in Australia (RRV, BFV, MVE) and worst for CHIKV, which had, to date, only been detected in travelers returning to Australia [31]. Intriguingly, there also appeared to be a better performance for the endemic alphaviruses that generally have a higher number of case counts, higher symptomatic attack rates, greater geographic distribution, and more regular seasonal variation in activity compared with MVE, which causes a very low proportion of notifiable and/or symptomatic cases and only sporadically causes outbreaks [31]. Such findings offer more evidence that Google-based surveillance is somewhat dependent on disease burden (which, in turn, likely prompts local population awareness of a particular arbovirus) and cyclical epidemiology. Importantly, Milinovich et al. examined the performance of Google-based surveillance of these pathogens on finer spatial scales and found that accuracy broadly deteriorated on a subnational scale.

Beyond arboviruses, predictive models fit with Google search terms have also been developed for malaria. Ocampo et al. fit a Google predictive model and compared with World Health Organization (WHO) reference malaria surveillance data for Thailand, with a high correlation between model-predicted and observed malaria activity (maximum *r* = 0.92) [11]. While this study was robust in that it used a holdout set of data to test predictions, it only provided predictions on the national level, again restricting its public health utility. Moreover, this study did not attempt to examine the role of seasonal effects and how much they may contribute to model performance, which may be considerable.

Finally, Google data have also been applied to the surveillance of a nontropical VBD, which primarily affects high-income settings. Seifter et al. qualitatively examined the agreement between US states’ volumes of Google searches for the term “Lyme disease” and US Centers for Disease Control and Prevention (CDC)-notified US case counts of Lyme disease over a 5.5-year period. Of the top 10 states for Google searches about Lyme disease, six were in the top 10 states with the highest Lyme disease incidence [32]. While this suggests some value as a surveillance signal, the study was limited in its scope, and it is hard to comment further about its utility for Lyme disease.

### Real-time surveillance of DENV with Twitter

Twitter is another promising large data source for VBD detection because user Tweets are geotagged to precise location, and an estimated 500 million daily Tweets are archived in real time [33]. In contrast with Google search logs, all Tweets are public, and extracting key Tweet words related to arboviruses has been explored as a method of VBD real-time detection. While Twitter does not aggregate normalized trends about specific Tweet terms, various application programming interfaces enable Twitter mining, and customized R packages allow the parsing of Twitter data in a biostatistical environment [34,35]. Tweets are often longer and have a more complex ontology than Google searches, so a variety of bioinformatic approaches have been required to dissect relevant terms from the corpus of a Tweet, thus posing a considerable technical challenge in using this data source. However, this also allows for capturing more personal Tweets, which would be more likely to represent true cases of a VBD rather than re-Tweets of a media or MoH announcement regarding disease activity. Aside from this, the concept of Twitter disease prediction is broadly similar to Google-based prediction, with time trends in Tweet volumes used as predictor terms in regression models that are fitted and validated against conventional-reference MoH DENV surveillance data. Such Twitter-based DENV models have been examined in Brazil, with two studies examining the performance of Twitter models in predicting DENV activity on a national and city scale against MoH data [36,37] and a third presenting a simple correlation of DENV-related Tweet volumes versus weekly DENV case counts in the state of Rio de Janeiro [38]. While the best performance of Twitter-based DENV surveillance was noted at a national aggregate scale (r = 0.98), and performance varied on finer spatial scales, Twitter does appear to offer remarkable spatial granularity. Souza et al. were able to track disease activity in locales as small as 100,000 persons. Importantly, the evaluation by Souza et al. also determined that disease incidence and population size generally influenced the accuracy of Twitter-based DENV surveillance on a city scale, although Twitter models still performed moderately well in some small-size and/or low-incidence cities. In their study, Gomide et al. also demonstrated how filtering Tweets to those specific to individual cases of DENV improved model performance [36].

### Wikipedia page views and DENV prediction

Wikipedia is a widely used, free, open-source, searchable encyclopedia and the fifth most visited website globally [39,40]. Trends in specific Wikipedia article views are provided via WikipediaTrends, with a granularity to the day and normalized as a function of all Wikipedia article views and searches for that time period [41]. Similar to the concept of Twitter and Google-based VBD surveillance, time series of Wikipedia topic views related to a specific communicable disease are thus obtainable and may serve as a proxy of that disease’s activity. Beyond capturing only a fraction of the search volumes of other big data sources like Google and Twitter, the primary limitation of WikipediaTrends is that it is unable to provide geographically defined page-view estimates [41]. However, other Wikipedia metadata sources allow limiting the searches to a specific language, thus indirectly determining trends in Wikipedia topic views by certain countries [42]. Generous et al. used this approach to examine the accuracy of Wikipedia-based predictive models in detecting changes in DENV and noted low to very low model prediction error in detecting monthly WHO-reported case count changes in Thailand (*r* = 0.86) and weekly case counts for Brazil (*r* = 0.93), although the lack of spatial resolution makes Wikipedia-based VBD surveillance quite limited for public health practice [6]. Moreover, it is unclear what proportion of this model’s predictive ability was accounted for by Google searches directing users toward the Wikipedia website. Since this study was conducted, Wikipedia has changed WikipediaTrends metrics to exclude such search engine redirects [41].

### Internet newswire scraping and crowd-sourced surveillance to enhance arbovirus surveillance

Informal reporting of epidemics and outbreaks through internet newswires has been demonstrated to be reasonably accurate and often more timely than official public health agency announcements [43,44]. The ProMed system was one of the earliest examples of scraping unstructured internet newswire data to rapidly detect outbreaks and epidemics [1]. The HealthMap project, launched in 2006, is an extension of this concept and provided an automated platform that offers outbreak and epidemic data signals scraped hourly from internet newswire feeds in addition to public health agency web alerts. In its current form, HealthMap draws data from four internet newswire streams (two English, two Chinese), the ProMed system, online official Eurosurveillance and WHO outbreak alerts, zoonosis outbreak databases, and the Geosentinel travel-illness network. [45]. Highly granular epidemic data are then mapped, real time, to GoogleMaps with a user-friendly graphical user interface. HealthMap therefore combines both conventional epidemiological and internet data streams in an automated, real-time fashion for a broad range of pathogens [45]. Importantly, unlike ProMed and other event-based news-scraping systems like WHO’s Global Public Health Intelligence Network, the HealthMap system may operate without any manual curation [46]. HealthMap also contains a more recent and semicurated function allowing for “crowd-sourced” epidemic surveillance (“Outbreaks Near Me”), a concept similar to the *Salud Boricua* mobile phone application, which enables digitally enrolled participants in Puerto Rico to report symptoms of DENV and CHIKV, irrespective of healthcare contact [47,48].

The performance of HealthMap in detecting DENV diffusion in the tropics was carefully evaluated by Hoen et al., who determined the sensitivity and specificity of HealthMap in detecting new DENV circulation in previously DENV-nonendemic regions in Latin America and used the CDC’s Yellow Book as a reference standard for DENV surveillance [49]. Over a 1.5-year period, HealthMap detected new autochthonous spread of DENV, with an overall sensitivity of 74% and specificity of 85% when compared to the CDC reference. This study also indicated the promising granularity of automated newswire scraping surveillance systems like HealthMap, with the detection of new DENV epidemics on a provincial scale. HealthMap’s DengueMap, a collaboration with the CDC, offers such granular surveillance to public health and clinical end users, including estimates of regions at risk for DENV spread. During the ZIKV epidemic, HealthMap has offered similar granular detail about locales with ZIKV circulation [50], and such information may be useful for personalized pretravel advice for those travelling to countries with a blanket label as “Zika endemic” yet which may have ZIKV-free areas.

Beyond disease detection, internet newswire scraping has also been explored for rapid determination of epidemic model parameters and even modeling the effect of interventions to inform public health preparedness and decision-making early in the course of VBD epidemics. Such an approach was demonstrated as feasible during the 2010 Haiti cholera and 2014–2015 West Africa Ebola epidemics [51] [52,53] and more recently was applied to the 2015–2016 ZIKV epidemic in Colombia. Majumder et al. sought to reconstruct the R_0_, predicted final outbreak size, and the duration of the ZIKV Colombia epidemic by using HealthMap data in conjunction with Google Trends time series data (for the search term “Zika”), the latter used to smooth incidence curves estimated by HealthMap data [54]. The confidence intervals of the R_o_ estimated from HealthMap and Google data overlapped with those estimated from formal MoH-reported ZIKV case counts. Digital data yielded similar estimates for the proportion of the susceptible population that would require vaccination, should a 100% efficacious vaccine have been developed before the end of the epidemic. This study made the point that important epidemic transmission parameters can be determined by using digital data alone should there be limited or no conventional surveillance infrastructure; however, it was unclear what proportion of HealthMap-measured ZIKV cases would be attributable to media reporting of Colombian MoH case counts or WHO case summaries.

More recently, HealthMap and ProMed data have been used alongside conventional surveillance data to reconstruct the dispersal of the ZIKV epidemic across Brazil and to map global environmental suitability for ZIKV, two notable examples of combining internet-based biosurveillance data with traditional surveillance data forms to enhance disease surveillance and prediction [55,56].

### Other internet data sources: YouTube and Facebook

While Facebook is the third most visited website in the world, its data are generally less amenable to enhance communicable disease surveillance because of the lack of public access to much of the data [40]. YouTube is open access and also commonly used, yet automating data extraction and content analysis from videos is challenging. As a result, this digital data stream has primarily been evaluated with respect to its potential to spread misinformation or clinically useful material rather than as an epidemic surveillance or prediction tool [57]. Nevertheless, particular mention should be made of the study by Alasaad, who sought to evaluate the surveillance potential of Facebook and YouTube posts concerning probable leishmaniasis cases in Syria [12]. While this study had a very limited validation and the reference data set was difficult to reproduce (Skype-calling individual clinicians in conflict zones with deteriorating public health infrastructure to confirm whether leishmaniasis had been seen in regions where YouTube and Facebook posts indicated disease), the findings were nevertheless valuable for exploring a possible public health application of social media in a conflict zone.

## Limitations, challenges and future directions in the application of internet-based VBD biosurveillance

The putative advantages of internet data for communicable disease surveillance are clear; namely, these data are free, fast, and may offer valuable surveillance signals in regions with limited conventional surveillance infrastructure and rising internet access. Such regions generally experience the brunt of VBD, although it is worth emphasizing that internet access may vary considerably between and within countries with high burdens of VBD [58]. The widespread use of HealthMap by international public health agencies, the partnership between the CDC and HealthMap’s DengueMap, the recent integration of internet-based DENV prediction platforms into broad nongovernmental organization (NGO) DENV initiatives [59], and the use of novel internet data streams in ZIKV predictions used to brief the US Government suggests that public health agencies and other key players are recognizing the potential of novel data sources in VBD epidemic response [27,60–62]. A recent pilot evaluation of US communicable disease surveillance professionals indicated that the majority were looking for new data sources to inform public health decision-making, and more specifically, that the majority found internet search and media scraping as moderately useful for early outbreak situational awareness [63].

Yet despite these promising examples, internet-based VBD surveillance arguably faces an unclear future in public health and clinical medicine. Despite over five years of academic publications, which often claim the importance of real-time digital data for VBD surveillance, and valuable conceptual frameworks, which suggest how digital data streams can be integrated into public health practice, there are little published data—to our knowledge—that have indicated how these novel forms of surveillance have actually been used in public health response or clinical medicine nor what measurable impact they have had on epidemic control or clinical care. Redressing this dearth of implementation science for these novel forms of VBD surveillance should be a core priority for this field (Box 1), particularly because feedback from end users of these methods would be invaluable for improving the development of future novel digital epidemiological tools for these and other pathogens and could form the backbone of a roadmap to implement these new technologies into public health practice across a range of settings. For example, we do not know if end users find the trade-off between accuracy and timeliness of internet data streams an acceptable one. Moreover, it is unclear if such end users find methods validated at coarse spatial or temporal scales at all useful for public health practice and whether they completely trust studies which have only externally validated the performance of predictive models over a short period of time (if at all), particularly given the important lessons learned regarding the deteriorating performance of Google Flu Trends over several years [21,64]. An evaluation of end users of an influenza A/H5N1 web-based expert epidemic intelligence system indicated that sensitivity and timeliness were perceived as the more important aspects of this type of internet-based zoonotic influenza surveillance approach, and similar efforts could be extended to end users of internet-based VBD surveillance methods [65].

**Box 1. Critical areas of research to improve the transition of internet-based biosurveillance into surveillance and control operations for VBDs**

- Meta-analysis of all published studies that have validated digital disease detection methods against reference public health data to determine the best estimates of accuracy of internet-based VBD surveillance by pathogen type, country income, internet access, and pathogen epidemiology.
- Implementation science studies to identify which sorts of digital data streams have actually been used during VBD epidemics, how they were used, and whether their timeliness had an impact on outbreak or epidemic control.
- Qualitative measurement of public health end user perceptions and sentiment regarding what features of digital disease detection systems are more important for practical use in VBD control, whether the systems are felt to be trustworthy, and how they would consider using them in VBD control.

Another key limitation to internet-based VBD surveillance has been the typical use of a Pearson correlation coefficient as a measure of instrument validity. While this does allow some kind of standardized comparison between the different types of data and would allow a valuable meta-analysis that could explore determinants of internet-based VBD surveillance performance, the Pearson correlation coefficient is highly prone to influential data points, makes an assumption that time series data points for communicable disease case counts are independent, and may be of limited use to public health end users who may be more interested in the early detection and prediction of VBD epidemic onset, epidemic peak, R_0_, total epidemic size, and other more practice-orientated quantitative surveillance goals [21,66]. Estimating these metrics takes statistical methods more sophisticated than mere Pearson correlation and will require extensive validation to ensure their accuracy [21]. The use of such metrics in evaluating internet-based DENV—and, more recently, ZIKV surveillance—indicates promising progress in this respect [14,54].

When considering the future role of internet big data in enhancing VBD surveillance and control, it is important to recognize that the developers of these digital surveillance methods have long cautioned that they are designed to augment rather than replace conventional surveillance data [3]. Efforts to combine digital data (e.g., Twitter and Google) with more conventional surveillance data (e.g., sentinel surveillance data from public health agencies) have proven fruitful for influenza surveillance [67]. This approach has been recently extended to ZIKV and DENV forecasting and/or nowcasting [55,68] and is perhaps one of the most promising future directions of internet-based surveillance for VBD and other pathogens. Along these lines, another promising opportunity is the use of internet data streams as a measurement of human behavior rather than as a proxy for disease incidence itself. A recent effort to clarify the drivers of the spatiotemporal dynamics the 2014 CHIKV outbreak in Martinique used just such an approach by using Twitter to measure local awareness and interest in protection against CHIKV, thereby allowing an evaluation of how such factors impact on the spread of this virus [69].

Finally, in another example of extrapolating advances in internet-based influenza surveillance to digital VBD surveillance, there have been very recent efforts to develop more flexible Google-based modeling frameworks in which regression models are continuously updated by using a sliding window training data set to best capture the changing association between population internet search behavior and DENV incidence, greatly improving prediction performance and offering the prospect of an automated forecasting system [68].

**Key learning points**

- There are several freely accessible internet data streams available to supplement traditional sentinel surveillance vector-borne viruses, parasites, and other pathogens, including ZIKV, DENV, other arboviruses, and malaria.
- The accuracy of these internet-based VBD surveillance systems varies. Spatial and temporal scale, disease burden, and seasonality are likely strong predictors of accuracy.
- User-friendly free platforms that combine digital and nondigital data streams to enhance VBD surveillance are now available for clinical and public health use.

**Top five papers**

- Gluskin RT, Johansson MA, Santillana M, Brownstein JS (2014) Evaluation of Internet-Based Dengue Query Data: Google Dengue Trends. PLoS Negl Trop Dis 8.
- Majumder MS, Santillana M (2016) Utilizing Nontraditional Data Sources for Near Real-Time Estimation of Transmission Dynamics During the 2015–2016 Colombian Zika Virus Disease Outbreak. 2: e30.
- Althouse BM, Scarpino SV, Meyers LA, Ayers JW, Bargsten M, Baumbach J, et al. (2015) Enhancing disease surveillance with novel data streams: challenges and opportunities. EPJ Data Sci 4(1), 17. https://doi.org/10.1140/epjds/s13688-015-0054-0, PMID: 27990325.
- Yang S, Kou SC, Lu F, Brownstein JS, Brooke N, Santillana M (2017) Advances in using Internet searches to track dengue. PLoS Comput Biol. 2017 Jul 20;13(7):e1005607
- McGough SF, Brownstein JS, Hawkins JB, Santillana M (2017) Forecasting Zika Incidence in the 2016 Latin America Outbreak Combining Traditional Disease Surveillance with Search, Social Media, and News Report Data. PLoS Negl Trop Dis. 2017 Jan 13;11(1):e0005295

## Supporting information

S1 File MEDLINE search ontology used to identify literature. (DOCX)
