# Supplementary material for: Internet-based biosurveillance methods for vector-borne diseases: Are they novel public health tools or just novelties?
Source: PLoS Negl Trop Dis. 2017 Nov 30;11(11):e0005871. doi: 10.1371/journal.pntd.0005871 (PMC5708615; doi:10.1371/journal.pntd.0005871)
Supplement: S1 File — (DOCX) [file pntd.0005871.s001.docx]

**MEDLINE search ontology**

(“Behavioral Risk Factor Surveillance System”[tiab] OR “Population Surveillance”[mh] OR “Population Surveillance”[tiab] OR “Epidemiological Monitoring”[mh] OR “Epidemiological Monitoring”[tiab] OR “Sentinel Surveillance”[mh] OR “Sentinel Surveillance”[tiab] OR surveillance[tiab] OR surveillance[mh] OR Biosurveillance [mh] OR biosurveillance[tiab] OR “disease surveillance”[tiab] OR “disease monitoring”[tiab] OR epidemiology[tiab] OR “Public health”[tiab] OR “Public health”[mh] OR “Public health Surveillance”[mh] OR trends[tiab] OR predict[tiab] OR prediction[tiab] OR tracking [tiab] OR nowcasting[tiab] OR forecasting[tiab] OR “attack rate”[tiab] OR incidence [tiab]) AND (“Communicable Disease”[tiab] OR “Communicable disease”[tiab] OR “Communicable diseases”[mh] OR infection [tiab] OR infections [tiab] OR “Infectious Diseases”[tiab] OR “Infectious Disease”[tiab] OR “Infectious Disease”[mh] OR infection[mh] OR outbreak[tiab] OR epidemic[tiab] OR outbreaks[tiab] OR epidemics[tiab] OR contagious[tiab] OR influenza[tiab] OR flu[tiab] OR virus[tiab] OR dengue[tiab]) AND (google[tiab] OR “search query”[tiab] OR “web query”[tiab] OR “web search”[tiab] OR “internet search”[tiab] OR “internet searches”[tiab] OR “online search”[tiab] OR “online searches”[tiab] OR “search engine”[tiab] OR “search engines”[tiab] OR ((web[tiab] OR online[tiab] OR internet[tiab]) AND (browser[tiab] OR browsers[tiab])) OR blog[tiab] OR blogs[tiab] OR blogging[tiab] OR OR crowdsourced[tiab] OR “digital detection” [tiab] OR “digital disease detection”[tiab] OR “internet based”[tiab] OR “web based”[tiab] OR twitter[tiab] OR tweet[tiab] OR tweets[tiab] OR “facebook”[tiab] OR yelp[tiab] OR wikipedia[tiab] OR yahoo[tiab] OR baidu[tiab] OR bing[tiab] OR “social media”[mh] OR “social media”[tiab] OR “internet news” OR “new media”[tiab]) NOT “Google Scholar”[tiab]
